# Mapping the landscape of PSC-CM research through bibliometric analysis

**DOI:** 10.3389/fcvm.2024.1435874

**Published:** 2024-10-10

**Authors:** Jun Li, Shangting Han, Fengxu Yu, Tao Li, Bin Liao, Feng Liu

**Affiliations:** ^1^ Key Laboratory of Medical Electrophysiology, Ministry of Education and Medical Electrophysiological Key Laboratory of Sichuan Province, Collaborative Innovation Center for Prevention of Cardiovascular Diseases, Institute of Cardiovascular Research, Southwest Medical University, Luzhou, China; ^2^ Department of Organ Transplantation, Department of Urology, Renmin Hospital of Wuhan University, Wuhan, China; ^3^ Department of Cardiovascular Surgery, The Affiliated Hospital of Southwest Medical University, Luzhou, China; ^4^ Cardiovascular Remodeling and Dysfunction Key Laboratory of Luzhou, Southwest Medical University, Luzhou, China

**Keywords:** PSC-CMs, organoids, organ-on-a-chip, maturation of cardiomyocytes, bibliometric analysis, visualization

## Abstract

**Objectives:**

The discovery of pluripotent stem cell-derived cardiomyocytes (PSC-CMs) has not only deepened our understanding of the pathogenesis and progression of heart disease, but also advanced the development of engineered cardiac tissues, cardiac regenerative therapy, drug discovery and the cardiotoxicity assessment of drugs. This study aims to visualize the developmental trajectory of PSC-CM research over the past 18 years to identify the emerging research frontiers and challenges.

**Methods:**

The literature on PSC-CMs from 2007 to 2024 was retrieved from the Web of Science and PubMed databases. Bibliometrix, VOSviewer and CiteSpace software were used for statistical analysis and visualization of scientific literature. Previous clinical trials were summarized using data from the ClinicalTrials.gov database.

**Results:**

A total of 29,660 authors from 81 countries and regions published 6,406 papers on PSC-CMs over the past 18 years. The annual output of PSC-CM research experienced a general upward trend from 2007 to 2021, reaching its peak in 2021, followed by a notable decline in 2022 and 2023. The United States has emerged as the most influential nation in this field, with Stanford University being the most prolific institution and Joseph C. Wu standing out as the most productive and highly cited scholar. Circulation Research, Circulation, and Nature have been identified as the most co-cited journals. Organ-on-a-chip, 3D bio-printing, cardiac microtissue, extracellular vesicle, inflammation, energy metabolism, atrial fibrillation, personalized medicine etc., with a longer burst period, and maturation of PSC-CMs, with the highest burst strength of 27.19, are the major research focuses for rigorous investigation in recent years. Cardiac organoid is emerging as a promising key research frontier. While the clinical trials of stem-cell-mediated treatment for heart diseases shows promise, significant challenges remain. Further research is imperative to optimize protocols, enhance cell delivery methods, and establish standardized practices to improve clinical outcomes.

**Conclusions:**

In conclusion, several major research hotspots, including engineered cardiac tissue and maturation, exosome-based regenerative therapy, inflammation response, energy metabolism, atrial fibrillation, and personalized medicine etc. will continue to attract substantial interest from investigators worldwide. Cardiac organoids to *in vitro* recapitulate the intricate human heart is emerging as a promising key research frontier. Significant challenges persist in the clinical trials of stem-cell-mediated therapies for heart diseases.

## 1 Introduction

Heart failure is the leading cause of hospitalization worldwide impacting over 64 million people. Despite significant progress in the diagnosis and management of this progressive cardiac condition, it continues to be a pressing global health concern (1). The primary cause of heart failure is cardiomyopathy, which can be classified into four types: dilated cardiomyopathy (DCM), hypertrophic cardiomyopathy (HCM), restrictive cardiomyopathy (RCM) and arrhythmogenic right ventricular cardiomyopathy/dysplasia (ARVC/D), based on morphological and functional criteria (2, 3). Several risk factors, including coronary artery disease, long-term high blood pressure, diabetes, obesity, binge drinking and genetic family history, contribute to the development of cardiomyopathy. To date, the most effective therapy option for this cardiac disorder remains unavailable due to a lack of patient-specific models of cardiomyopathies that can accurately and comprehensively recapitulate the disease phenotype and significantly deepen our understanding of the etiology, allowing for the identification of new therapeutic targets and drug discovery (4–6).

In 2006, mouse induced PSCs were first generated via overexpression of a set of specific transcription factors (7). In 2007, Kazutoshi Takahashi et al. demonstrated that human induced PSCs can differentiate into cardiomyocytes *in vitro*. It was not until 2009 that Zhang et al. provided the first evidence of functional cardiomyocytes derived from human induced PSCs. This ground-breaking discovery holds immense potential for cardiac regenerative therapies, disease modeling, high-throughput screening in drug discovery, and personalized or population-based toxicity assays considering ethnicity and genetic variations (8–10). Recently, the application of three-dimensional (3D) tissue engineering and CRISPR/Cas9 genome editing techniques in induced PSC-CM research has significantly accelerated progress in the field of cardiac tissue engineering (11–14). However, human induced PSC-CMs resemble more fetal or neonatal cardiomyocytes, appearing as small cell size, short sarcomeres (1.6 µm), T-tubule absence, lack of some ion transporters and regulatory proteins in the sarcoplasmic reticulum, lower mitochondrial crista density, higher resting membrane potential of −50 to −60 mV, and predominant glycolysis for energy production. The structural and functional immaturity of PSC-CMs poses a significant obstacle to their widespread application (15, 16).

In this study, we utilized bibliometric tools to quantitatively analyze and visualize the research on PSC-CMs from 2007 to 2024. The objective is to evaluate the present state of global research on PSC-CMs, identify research issues and frontiers, and provide investigators with valuable insights into potential future research directions in the field of PSC-CMs.

## 2 Methods

### 2.1 Data collection and cleaning

The SCI-Expanded Web of Science Core Collection (WoSCC), including over 12,000 academic journals, is recognized as one of the world's most comprehensive database platforms (17). We accessed this platform to retrieve scientific literature and collected global academic information for a full bibliometric analysis. In 2007, it was first evidenced that human induced PSCs can be successfully differentiated into cardiomyocytes *in vitro* (18), therefore, we selected 2007 as the beginning year for our retrieval process.

**Figure 1:**
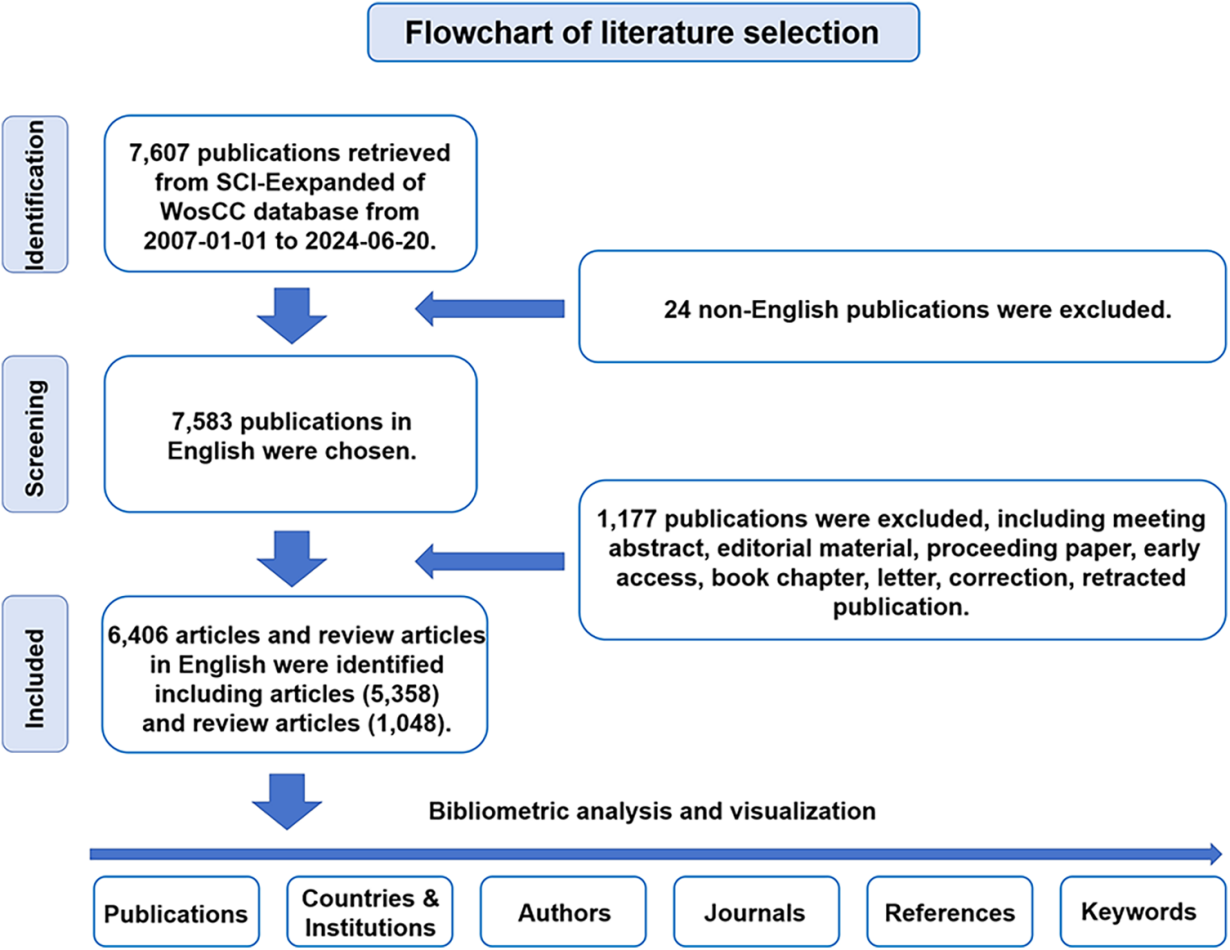
Flowchart of literature selection.

For WoSCC retrieval, we accessed the SCI-Expanded WoSCC to conduct a literature retrieval from January 1, 2007 to June 20, 2024. The retrieval strategy was as follows: [TS = (cardiomyocyte*) OR TS = (“cardiac myocyte*”) OR TS = (“cardiac muscle cell*”) OR TS = (“cardiac cell*”) OR TS = (“three germ layers in vitro*”)] AND [TS = (“induction of pluripotent stem cell*”) OR TS = (“induced pluripotent stem cell*”) OR TS = (“pluripotent stem cell*”) OR TS = (“embryonic stem cell*”)]. Only “articles” and “reviews” in English were extracted. The other approximately eight document types were excluded through the filters of WoSCC. All searches were performed on a single day, June 20, 2024. Documents were categorized based on species-specific pluripotent stem cells using the filter of SCI-Expanded Web of Science Core Collection. Documents were downloaded as original data from the SCI-Expanded WoSCC for further bibliometric analysis (Figure 1). Statistical analysis indicators mainly include the number of publications and citations, journals, publication year, countries/regions, institutions, authors, keywords and references. The different names representing the same country or institute were merged. Duplicate words or noun phrases were merged into one word or one noun phrase using a synonym database file, all misspelled elements were corrected and all useless words were excluded before data analysis by Bibliometrix, CiteSpace and VOSviewer.

For PubMed retrieval, the retrieval strategy was as follows: ((“Pluripotent Stem Cells"[Mesh]) OR (“Pluripotent Stem Cell*"[tw])) AND ((((((“Myocytes, Cardiac"[Mesh]) OR (“Cardiac Myocyte*"[tw])) OR (“Cardiac Muscle Cell*"[tw])) OR (“Heart Muscle Cell*"[tw])) OR (Cardiomyocyte*[tw])) OR (“three germ layers *in vitro*"[tw])). Time span is from January 1, 2007 to June 20, 2024.

For the clinical trial retrieval on the ClinicalTrials.gov database, the retrieval strategy was as follows: [(stem cell*) OR (progenitor cell*)] AND [(cardiac disease*) OR (heart disease*)]. Time range: January 1, 2007 to June 20, 2024.

### 2.2 Scientometric analysis

In this study, the bibliometric online analysis platform (bibliometric.com), CiteSpace (version 6.3.R3) and VOSviewer (version 1.6.20) were used to statistically analyze the scientific literature. The parameters of CiteSpace are as follows: (1) Time slicing is from January 1, 2007 to June 20, 2024, and the time cutoff point of analysis is one year; (2) g-index (*k* = 25 or 100); (3) Pruning: Pathfinder and Pruning sliced networks are selected to improve the calculation efficiency and trim the redundant links; (4) Link strength: Cosine; Link scope: Within slices; (5) Minimum duration (2 for keywords; 5 for references). In VOSviewer, we set up the minimum number of publications or citations or occurrence frequencies for the nodes based on the needs of effective data visualization when mapping the cooperation of countries/regions or institutions or authors, and the co-occurrence of keywords.

## 3 Results

### 3.1 Annual quantitative distribution of publications

**Figure 2:**
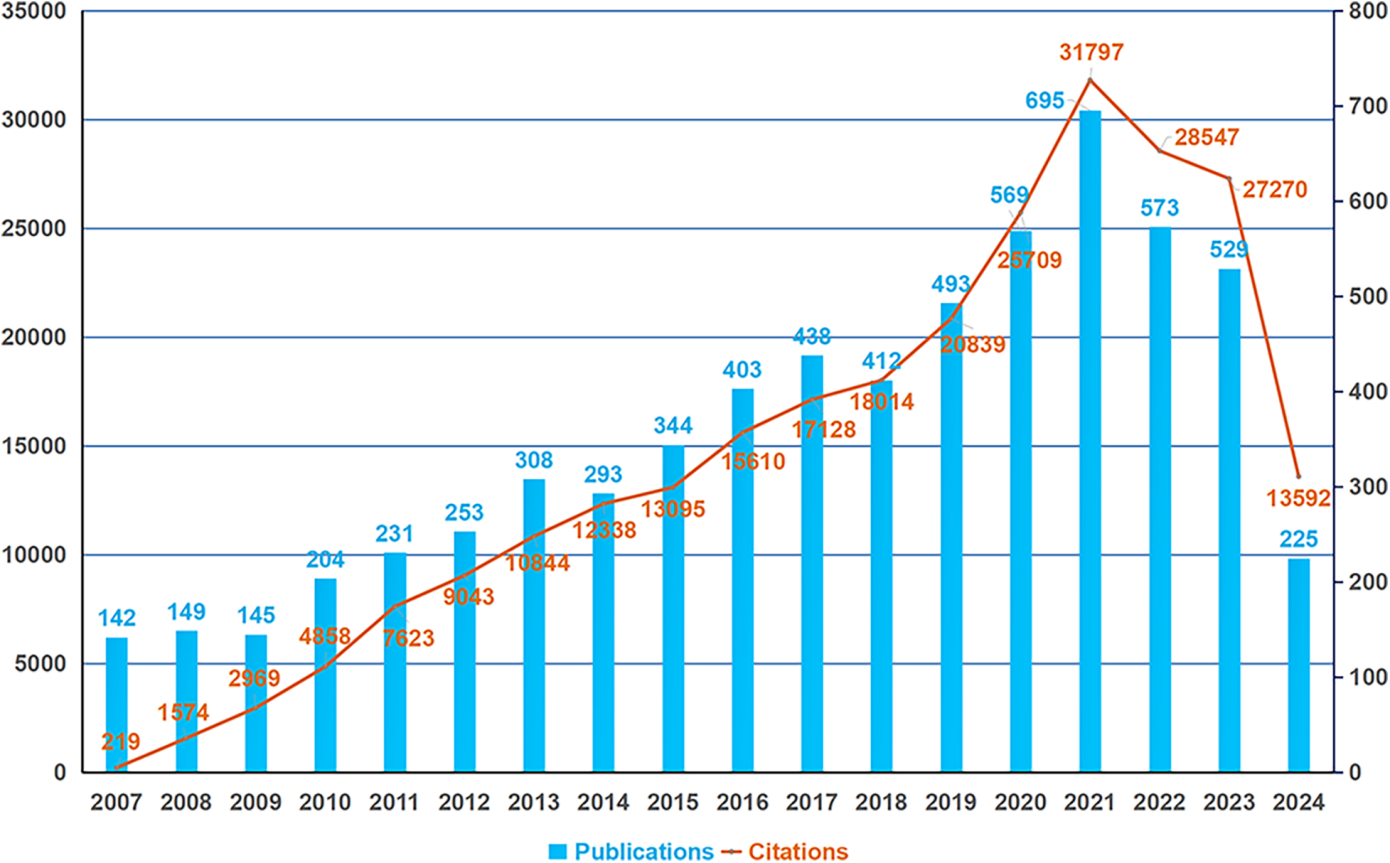
Global trend in the annual outputs and citations of publications on PSC-CMs from 2007 to 2024. The horizontal axis denotes the publication year, with the blue bar indicating the annual publication outputs and the orange curve representing the annual total citations.

The variation in scientific publication output over time reflects a research field's evolutionary trajectory. Figure 2 illustrates the annual volume and citations of publications concerning PSC-CMs over the past 18 years. From 2007 to 2021, global publications on PSC-CMs displayed an overall upward trend, peaking in 2021 with 695 papers before declining in 2022 and 2023 with declines of 17.6% and 23.9% respectively, marking the major advance in PSC-CM research before 2021. This recent decrease is likely attributable to some challenges such as PSC-CM immaturity, poor engraftment, and tumorigenic/immunogenic risks, which are hampering the clinical translational application of PSC-CMs. To ensure sustained advancement in the PSC-CM field, further investigation is essential to address these limitations (19–21).

### 3.2 Contribution of countries and institutions

**Figure 3:**
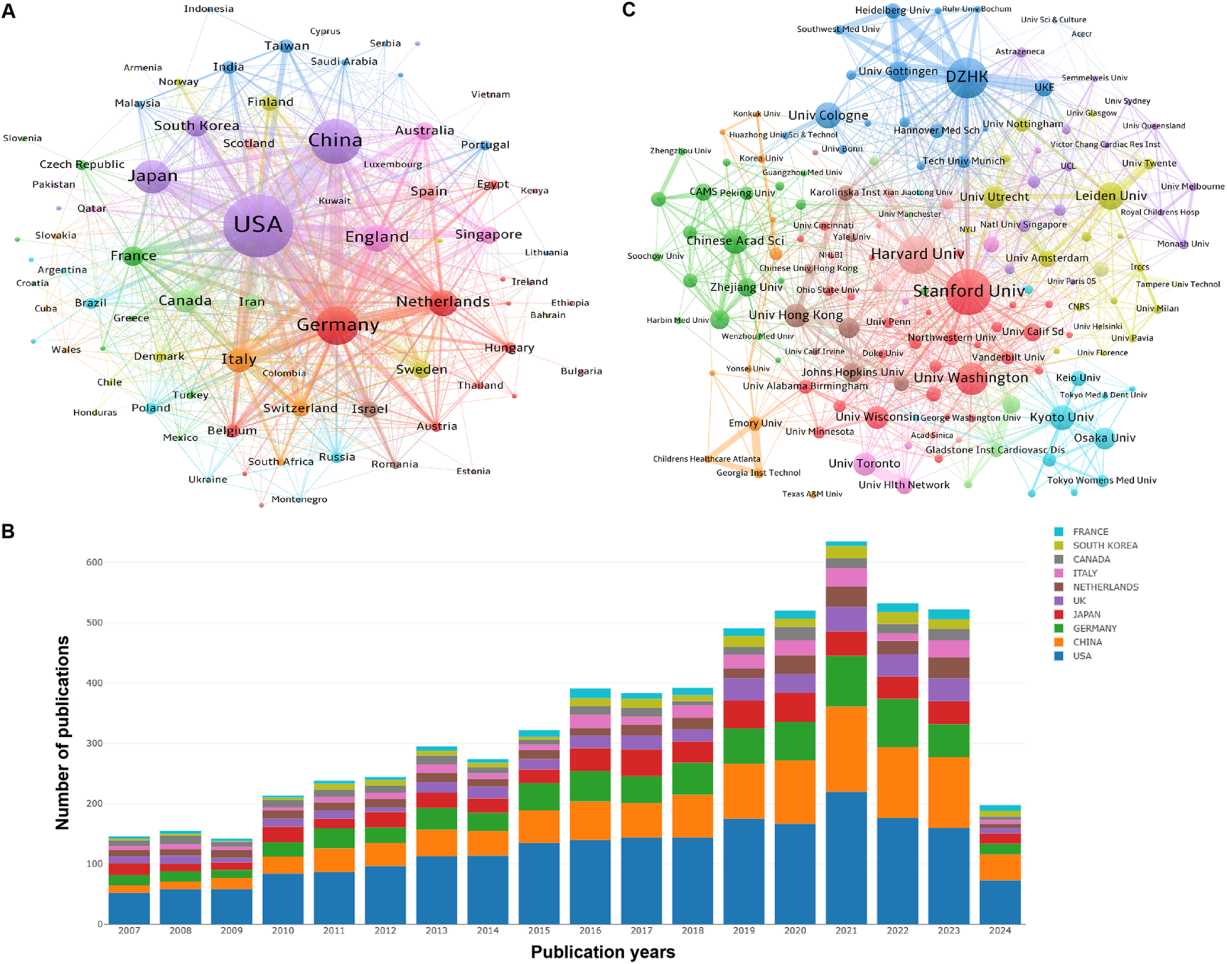
Collaboration and contribution of countries/regions or institutions in the field of PSC-CMs. **(A)** Collaboration network of countries/regions (publications per country ≥ 1). **(B)** the annual volume changes of publications in the top 10 countries from 2007 to 2024. **(C)** Collaboration network of institutions (publications per institution ≥ 15). The nodes in these networks represent countries/regions or institutions, with the larger node corresponding to more publications. The lines connecting these nodes indicate academic cooperation, wherein a thicker line corresponds to a more intimate partnership. The different colors of nodes and lines indicate different collaborative clusters, which were automatically calculated by VOSviewer.

The co-authorship of countries (or institutions) involved in the papers on PSC-CMs was analyzed using VOSviewer. From 2007 to 2024, a total of 81 countries and regions published 6,404 papers on PSC-CMs. In terms of academic collaboration among countries (Figure 3A and Table 1), the United States exhibits the highest total link strength (1,671 times), indicating strong international cooperation with other countries, followed by Germany (896), China (691) and England (612). In the cooperation network, the intermediate centrality score is utilized to measure the effectiveness of an entity in disseminating information to other entities within the network. In general, an entity with a higher centrality acts as the key hub to connect various clusters. Upon analyzing the intermediate centrality, it becomes evident that the United States, Germany, China, England, and Netherlands possess a higher score of more than 0.1, suggesting these countries play a core role as the vital bridge within the national cooperation network in the field of PSC-CMs. The United States and Germany are the two most influential nations, leading the research of PSC-CMs. Among the top 10 prolific countries (Figure 3B and Table 1), the United States ranks first with 2,683 publications, accounting for 41.90% of the total, followed by China with 1,211 publications (18.91%) and Germany with 859 publications (13.41%). Scientific impact refers to the extent to which scholar's articles have contributed to knowledge and influenced the thoughts of others (22). Citation impact is a crucial metric for assessing a publication's scientific influence (23). The top 4 countries with the highest total citation are the United States (154,247 citations), Japan (37,553), Germany (31,122) and China (28,786).

**Table 1:** Top 10 prolific countries in the field of PSC-CMs.

Rank	Country	Article counts	Citations	ACI	Centrality	TLS
1	USA	2,683	154,247	57.49	0.25	1,671
2	China	1,211	28,786	23.77	0.15	691
3	Germany	859	31,122	36.23	0.25	896
4	Japan	626	37,553	59.99	0.05	280
5	England	407	18,517	45.49	0.13	612
6	Netherlands	392	18,668	47.62	0.12	491
7	Italy	308	11,520	37.40	0.09	473
8	Canada	301	16,667	55.37	0.04	289
9	South Korea	226	5,730	25.35	0	155
10	France	187	5,838	31.22	0.07	274

Note: TLS, total link strength; ACI, average number of citations per publication.

The top 10 prolific institutions are presented in Table 2. Stanford University contributes the maximum number of publications (297), followed by the German Center for Cardiovascular Research (DZHK) (283), Harvard University (243) and Leiden University (155). We analyzed the cooperation of 217 institutions with at least 15 papers. Figure 3C shows the collaborative network of institutions, highlighting 6 key collaborative clusters represented by Stanford University, DZHK, Harvard University, Leiden University, Chinese Academy of Sciences, Kyoto University and Osaka University. However, a majority of institutions demonstrate an intermediate centrality of less than 0.1, unveiling that the studies on PSC-CMs that had been conducted by different institutions remain somewhat isolated, which underscores the need to enhance academic collaboration across institutions in the future.

**Table 2:** Top 10 productive institutions in the field of PSC-CMs.

Rank	Institution	Article counts	Centrality	TLS
1	Stanford Univ	297	0.05	352
2	DZHK	283	0.02	523
3	Harvard Univ	243	0.08	388
4	Leiden Univ	155	0.02	212
5	Univ of Toronto	151	0.02	116
6	Chinese Academy of Sciences	150	0.11	181
7	Univ of Cologne	146	0.06	97
8	Univ of Washington	144	0.02	164
9	Kyoto Univ	135	0.06	133
10	Univ of Gottingen	131	0.02	261

### 3.3 Contribution of scholars

**Figure 4:**
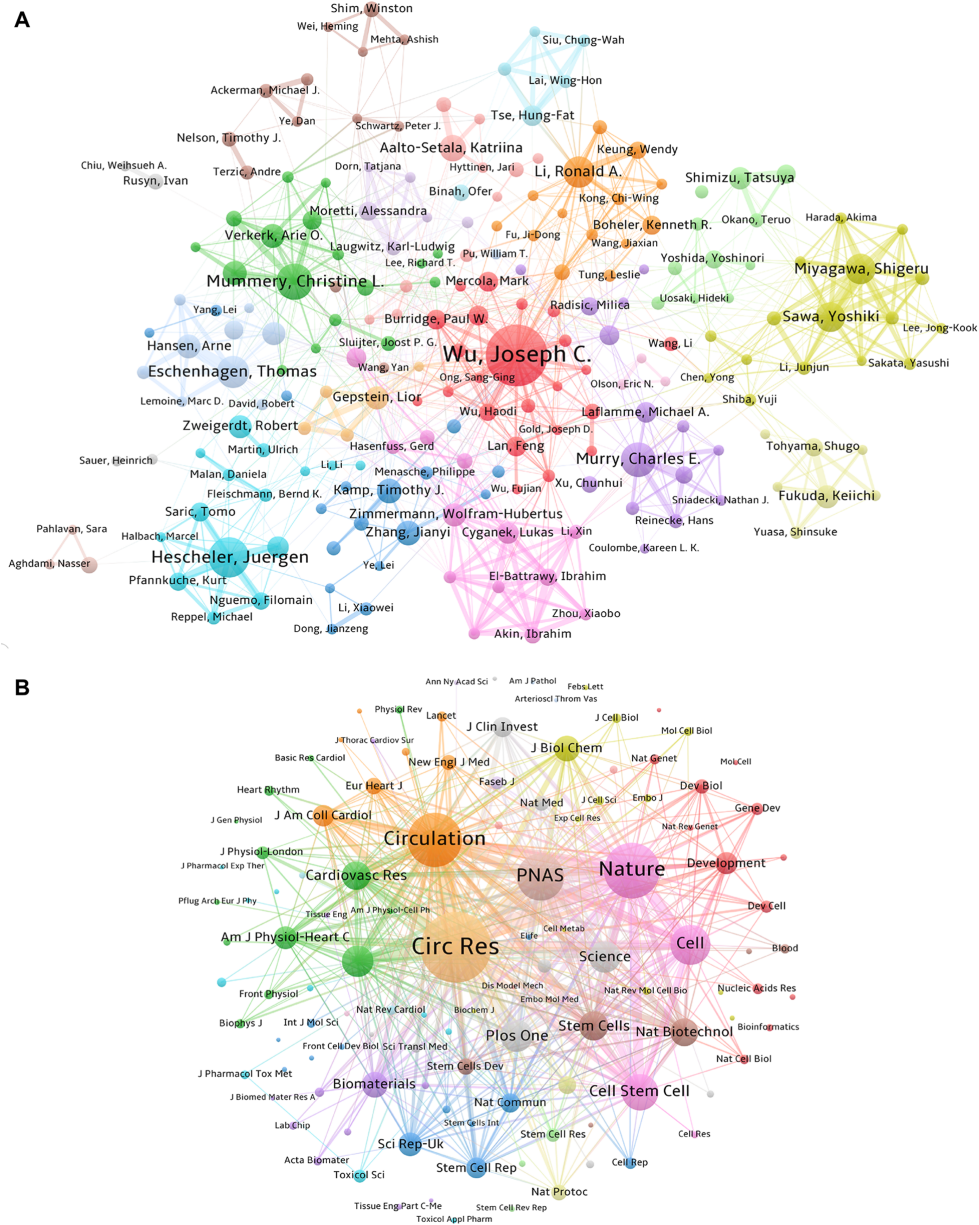
Collaboration and contribution of authors and the core co-cited journals. **(A)** Author collaboration network based on the co-authorship of papers related to PSC-CMs (articles per author ≥ 15). The sizes of nodes correspond to the number of papers. The links indicate the existence of a cooperative relationship among the authors, with the thicker line corresponding to a closer partnership. Authors within the same cluster exhibit a higher degree of collaboration. **(B)** Network of co-cited journals (citations per co-cited journal ≥ 400). The size of the nodes represents the co-cited frequencies of journals. The link reflects the co-cited relationship between the journals, with a thicker line indicating a higher co-cited frequency, which can be weighted by a quantitative indicator TLS.

The research output of scholars can be measured using both the publication volume and the citation frequency. Highly cited scholars have their studies widely recognized and cited by other investigators in the same field, reflecting the significant impact of their research on shaping the developmental trajectory of one field and advancing our understanding. A total of 213 out of 29,660 authors worldwide performing the investigation of PSC-CMs had published at least 15 papers (Figure 4A). The top 10 authors who have made substantial contributions to the field are presented in Table 3. Joseph C. Wu is the most prolific scholar, with 167 publications and 75.26 average citations per paper (ACI), followed by Juergen Hescheler (95; 29.86), Christine L Mummery (82; 90.99), Charles E Murry (77; 186.12), and Thomas Eschenhagen (70; 65.93). Notably, Charles E Murry, Timothy J Kamp, Christine L. Mummery, and Joseph C. Wu shine as the top four highest average cited authors with ACI scores of 186.12, 146.64, 90.99 and 75.26 respectively.

**Table 3:** Top 10 prolific authors in PSC-CM research.

Rank	Author	Institutions	Article counts	Citations	ACI	Centrality	TLS
1	Joseph C Wu	Stanford Univ.	167	12,568	75.26	0.40	317
2	Juergen Hescheler	Univ. of Cologne	95	2,837	29.86	0.08	165
3	Christine L Mummery	Leiden Univ.	82	7,461	90.99	0.11	171
4	Charles E Murry	Univ. of Washington	77	14,331	186.12	0.07	142
5	Thomas Eschenhagen	UKE	70	4,615	65.93	0.06	146
6	Ronald A Li	Univ. of Hong Kong	68	3,317	48.78	0.07	157
7	Shigeru Miyagawa	Osaka Univ.	65	1,385	21.31	0.03	252
8	Yoshiki Sawa	Osaka Univ.	63	1,464	23.24	0.03	240
9	Katriina Aalto-setala	Tampere Univ.	55	1,795	32.64	0.02	58
10	Timothy J Kamp	Univ. of Wisconsin	50	7,332	146.64	0.07	71

By analyzing the co-occurrence pattern of authors, we can acquire valuable insights into collaborative pattern, intellectual connection, and author impact in a specific research field. This information not only enhances our understanding of the field's knowledge flow and intellectual structure but also aids to identify influential author and potential collaborator. Figure 4A shows 9 major collaborative clusters represented by these core authors in the field of PSC-CMs, including Joseph C. Wu, Juergen Hescheler, Christine L Mummery, Charles E Murry, Thomas Eschenhagen, Ronald A Li, Shigeru Miyagawa, Wolfram-Hubertus Zimmermann and Timothy J Kamp. However, it is notable that not all authors hold equal weights; only Joseph C. Wu and Christine L Mummery have an intermediate centrality score greater than 0.1 (Table 3), revealing their prominent involvement in the cooperative network.

### 3.4 Core journals

Over the last 18 years, 896 journals have published studies on PSC-CMs. As presented in Table 4, Circulation Research has the highest average citation rate of 113.59, followed by Stem Cells (56.98) and Stem Cell Rep (42.4). To determine the core journal in the field, we mapped the co-citation relationship of journals using VOSviewer, spanning the period from 2007 to 2024 (Figure 4B). Among the top ten co-cited journals (Table 4), Circulation Research stands out with 19,100 citations and a total link strength of 1,246,825 times, followed by Nature (13,968; 882,289) and Circulation (13,548; 877,313). These three journals are highly prestigious and have a significant academic impact in the field of PSC-CMs based on their highly cited frequencies and impact factors.

**Table 4:** Top 10 productive journals and co-cited journals related to PSC-CMs.

Rank	Journal	Paper counts	ACI	IF (2024)	Co-cited journal	Citations	TLS	IF (2024)
1	Stem Cell Res	231	13.00	0.8	Circ Res	19,100	1,246,825	16.5
2	Plos One	200	38.23	2.9	Nature	13,968	882,289	50.5
3	Sci Rep	197	27.89	3.8	Circulation	13,548	877,313	35.5
4	Int J Mol Sci	151	10.84	4.9	PNAS	11,281	706,707	9.4
5	Stem Cell Rep	147	42.40	5.9	Cell	9,092	570,278	45.5
6	Circ Res	139	113.59	16.5	Cell Stem Cell	8,038	525,634	19.8
7	Stem Cells	123	56.98	4.0	Plos One	6,867	426,975	2.9
8	J Mol Cell Cardiol	119	42.36	4.9	J Mol Cell Cardiol	6,676	464,012	4.9
9	Stem Cell Res Ther	102	17.38	7.1	Science	6,669	427,819	44.7
10	Stem Cell Dev	99	36.65	2.5	Stem Cells	6,159	354,013	4.0

### 3.5 Research hotspots and trends

#### 3.5.1 Reference co-citation analysis

**Figure 5:**
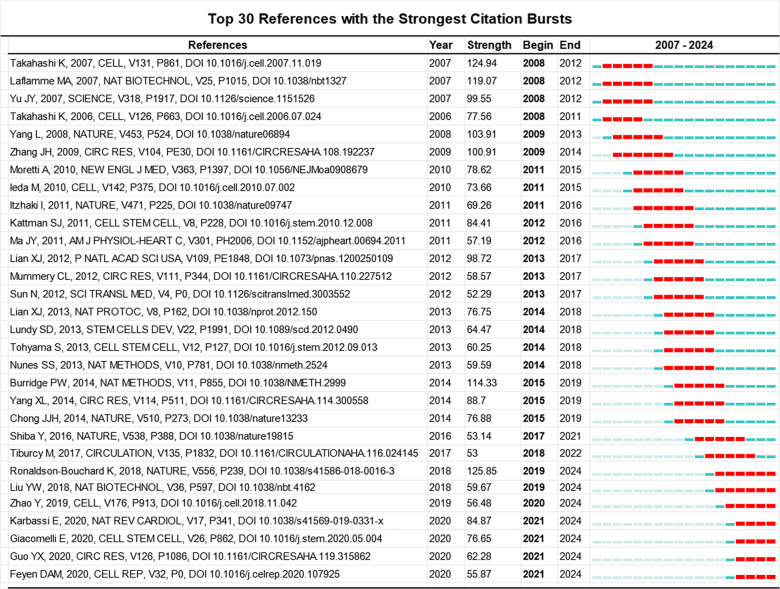
Top 30 references with the strongest citation bursts involved in the research of PSC-CMs. Note: the red bar stands for the duration of citation burst for the references.

The representative co-cited references are the cornerstone in a research field, reflecting the discipline's development history and influential perspectives (24). The citation burst is defined as a sudden surge in citations in certain periods, indicating a significant increase in research interest within specific themes (25). In Figure 5, we list the top 30 references with the strongest citation burst. The earliest reference was published by Takahashi et al. (18), and first displayed that human iPSCs can differentiate into cardiomyocytes *in vitro*. This article experienced a burst duration of 5 years, from 2008 to 2012, with a burst strength of 124.94. Subsequently, Zhang et al. (26) first successfully demonstrated the potential of human induced PSCs to *in vitro* differentiate into the functional cardiomyocyte. Its citation burst lasted 6 years from 2009 to 2014 with a burst strength of 100.91. In 2010 (27), Moretti et al. published their work on patient-specific induced PSC models for long-QT syndrome, which was the first application of human induced PSC-CM in the disease modeling. This article experienced a burst duration of 5 years from 2011 to 2015, with a burst strength of 78.62. Over the next two years (2012–2013), Lian et al. (28) and Tohyama et al. (29) explored the methodology for the purity and scalable production of human induced PSC-CMs. Their citation bursts lasted 5 years. Furthermore, Chong et al. (30), Shiba et al. (21) and Liu et al. (31, 32) investigated the potential of human PSC-CMs to regenerate non-human primate hearts. Recently, the most frequently cited topic was the maturation of human PSC-CMs. Ronaldson-Bouchard K, Giacomelli E and Feyen DAM are 3 core investigators in this research theme (13, 33, 34).

**Figure 6:**
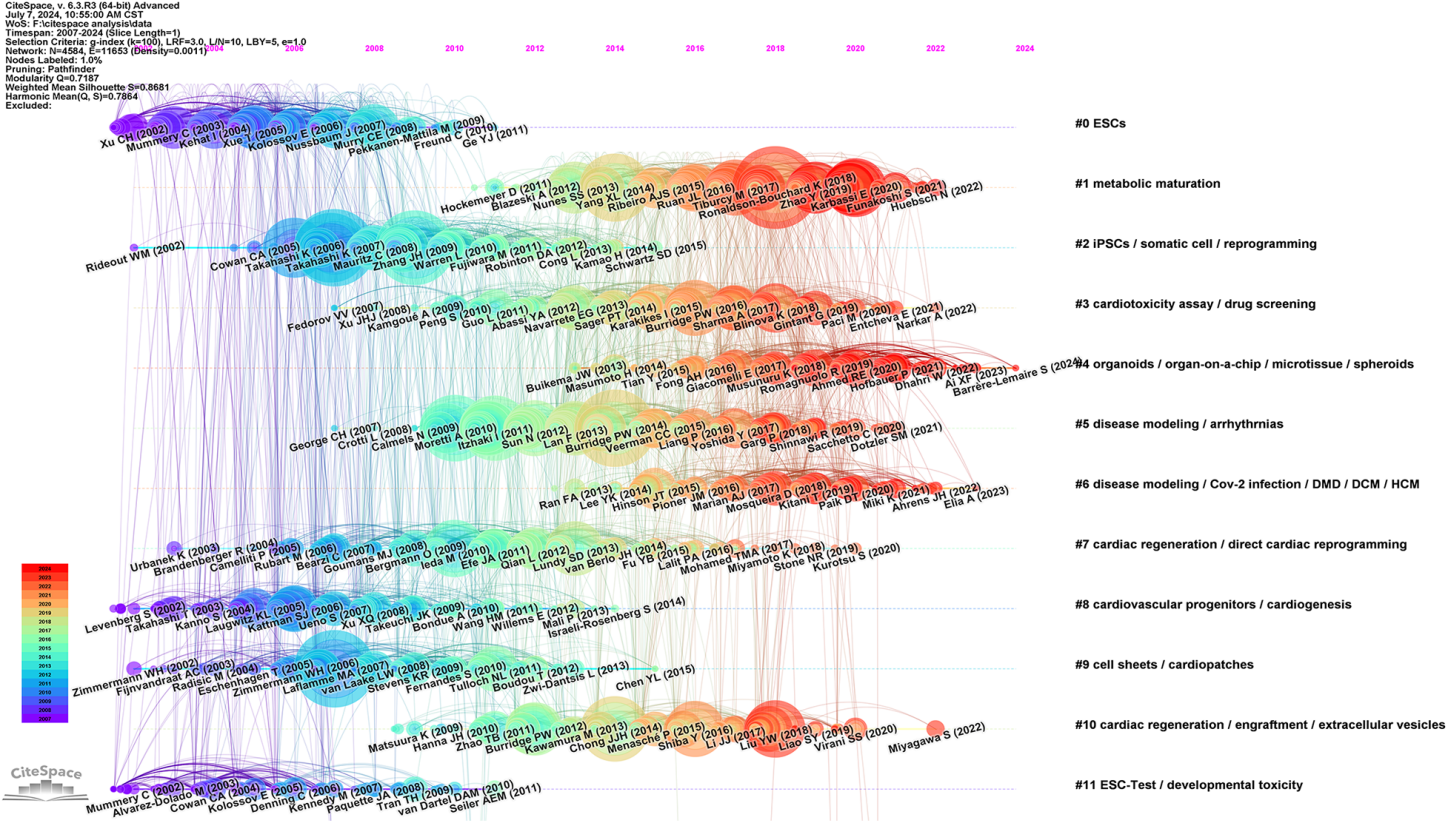
Timeline view of co-cited references related to PSC-CMs. A timeline view of the top 12 clusters with different research topics in the co-cited reference network displays the distribution, chronological span and trends of various research themes in the field. Each horizontal row represents a cluster, while each tree ring node along the row indicates a cited paper. Citation tree rings reflect the citation history of an article. The color of a ring represents the time of corresponding citations. The thickness of a tree ring is proportional to the number of citations in a given time slice. The node size denotes the frequency of the paper's co-citation. The node's position designates the publication year of the corresponding articles. Nodes experiencing citation bursts are spotlighted with red dots or circles. Cluster terms are arranged vertically on the right in descending order of cluster size. Lines connecting two different nodes symbolize the co-citation relationship between two studies. Modularity *Q* = 0.7187; weighted mean silhouette *S* = 0.8681.

To determine the temporal evolution of the knowledge structure in the field of PSC-CMs, we utilized CiteSpace to generate a timeline view of the co-cited reference network. Figure 6 shows the arrangement of references on the horizontal timeline row based on their publication year and co-citation frequencies. Each row corresponds to a specific cluster. Among the top 11 clusters, the most recently active ones are Cluster 1 (metabolic maturation), Cluster 3 (cardiotoxicity assay/drug screening), Cluster 4 (organoids/organ-on-a-chip/microtissue/spheroids), Cluster 5/6 (disease modeling/Cov-2 infection/DMD/DCM/HCM/arrhythrnias), and Cluster 7/10 (cardiac regeneration and repair). These most recently active clusters highlight the current research hotspots and emerging trends in the field. Cluster 1, representing the theme of engineered cardiac tissues and their maturation, will continue to receive significant attention from researchers worldwide. The cardiac organoids (Cluster 4) is expected to become a potential research hotspot in the near future; while Cluster 3 (cardiotoxicity assay/drug screening), Cluster 5/6 (disease modeling/Cov-2 infection/DMD/DCM/HCM/arrhythrnias), and Cluster 7/10 (cardiac regeneration and repair) have consistently been major themes of investigation throughout the entire development of PSC-CM research. They span a longer period and continue to remain active at present. In contrast, highly cited studies in Clusters 0 (ESCs), Cluster 8 (cardiovascular progenitors/cardiogenesis), Cluster 9 (cell sheets/cardiopatches) and Cluster 11(ESC-Test/developmental toxicity) have been dwindling in recent years.

#### 3.5.2 Keyword co-occurrence and citation bursts

Keywords in scientific literature serve as representative terms that provide a concise overview of the research theme. Co-occurrence analysis of keywords and citation burst detection are two extensively utilized bibliometric methods (35). The former provides valuable insights into the relationships between various research themes, allowing researchers to determinate emerging interdisciplinary topics and potential collaborations. The latter tracks the evolution of research trends over time, identifies frontier and hotspots, and recognizes landmark articles that significantly impact the scientific community, thereby shaping the direction of a research field.

**Figure 7:**
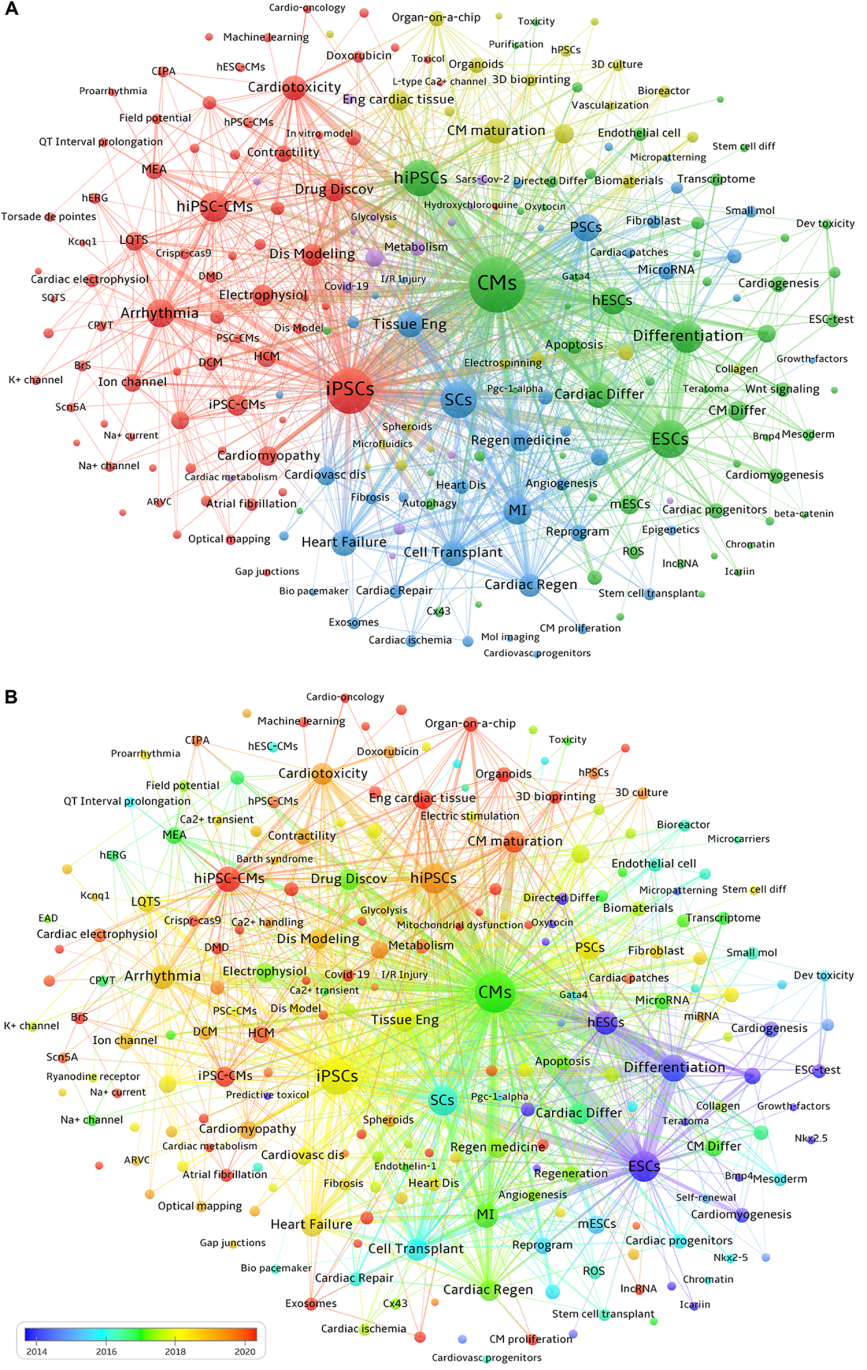
Co-occurrence and distribution over time of keywords. **(A)** Each node represents a keyword. The size of the node indicates the occurrence frequency of a keyword, and the larger the node is, and the higher its corresponding occurrence frequency. Keywords with close correlation are grouped into one cluster with the same color. The thickness of the connecting line between nodes reflects the strength of co-occurrence, with a thicker line indicating a higher co-occurrence frequency of the two connected keywords. **(B)** Distribution of keywords according to the average year of publication. Blue nodes signify earlier-emerging keywords, around or before 2015, green nodes represent keywords appearing approximately 2017, and orange-red nodes denote frequently occurring keywords around or after 2020.

First, we employed VOSviewer to extract keywords that appear at least 10 times from a total of 6,406 publications for clustering analysis of keyword co-occurrences. As presented in Figure 7A, seven main clusters represented by different colors, reflecting the 4 major research directions in the field of PSC-CMs, are identified. The yellow-green cluster reflects the investigation of engineered cardiac tissues and their maturity, including these keywords, such as Engineered Cardiac Tissue, Cardiomyocyte Maturation, Electric Stimulation, Stiffness, Biomaterials, bioreactors, Bioengineering, Microfluidics, 3D Bioprinting, Organ-On-A-Chip, Organoids, etc. The red cluster highlights the application of hiPSC-CMs in disease modeling and drug discovery, involving in keywords such as Disease Modeling, Arrhythmia, DMD, HCM, DCM, Drug Discovery, Drug Testing, Predictive Toxicology, Safety Pharmacology, Cardiotoxicity, Personalized Medicine, etc. The blue cluster indicates the research on cardiac regeneration, containing keywords such as Myocardial Infarction (MI), Cardiac Ischemia, Cardiac Regeneration, Cell Transplantation, Extracellular Vesicles, Exosomes, Direct Reprogramming, PSCs, etc. The green cluster represents the investigation into the directed differentiation, including Directed Differentiation, ESCs, hESCs, PSCs, hiPSCs, Cardiac Progenitors, Cardiomyocyte Differentiation, Retinoic Acid, Wnt Signaling, Bmp4, Ventricular Cardiomyocytes, SAN, Proliferation, Purification, etc. The purple cluster reflects numerous studies on the etiology and mechanisms of heart diseases, encompassing these keywords such as Metabolism, Mitochondrial Dysfunction, Oxidative Stress, Inflammation, Cytokines, Covid-19, Sars-Cov-2, RNA-seq, etc.

VOSviewer software can be used to visualize the chronological view of keyword co-occurrence by assigning different colors to each included keyword based on its average appearance year. In Figure 7B, the color of each node indicates the average appearance year of a keyword. According to the color gradient bar, blue-yellow nodes represent keywords that appeared relatively earlier, before 2019. While red nodes indicate keywords that have been the focuses of intensive investigation in recent years, including Organoids, Organ-on-a-chip, Engineered Cardiac Tissues, Cardiac Microtissues, Maturation of hiPSC-CMs, 3D Bioprinting, SARS-CoV-2, Inflammation, Extracellular Vesicles, Exosomes, Energy Metabolism, Personalized Medicine, etc.

**Figure 8:**
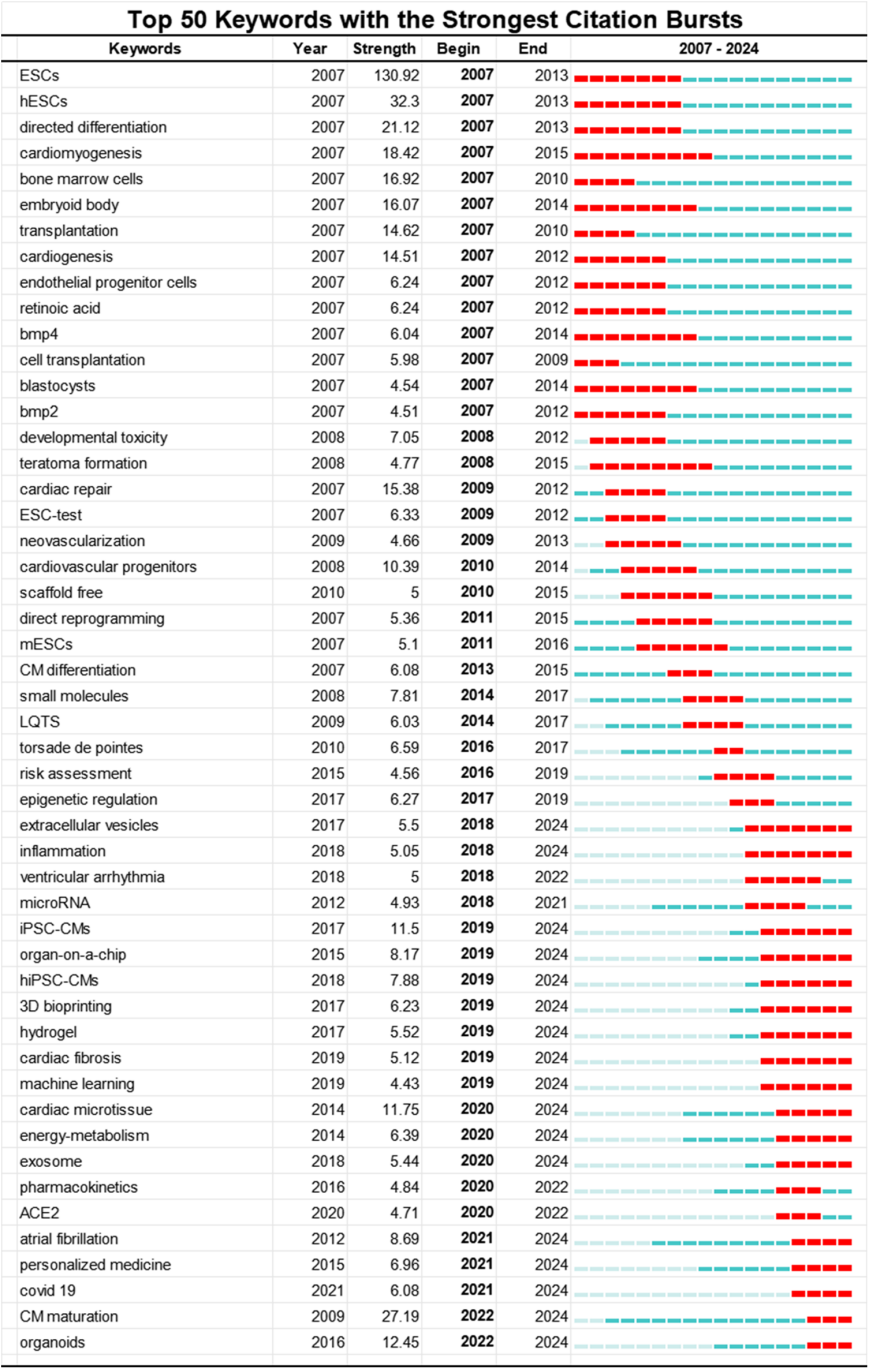
Top 50 keywords with the strongest citation bursts involved in the research of PSC-CMs. The red bar stands for the duration of citation burst for keywords.

Third, we utilized Kleinberg's burst detection algorithm in CiteSpace to analyze the top 50 keywords with the highest burst strength, aiming to detect current hot topics in the field of PSC-CMs. Among these keywords shown in Figure 8, we specifically focused on those that started bursting after 2018, allowing us to gain insights into the research frontiers. Excluding the search keywords human induced PSC-CMs and induced PSC-CMs, we identified 18 hot-spot keywords with the strongest citation burst from 2018 to 2024. Extracellular Vesicles and Inflammation exhibited burst periods exceeding 6 years, while Organ-on-a-chip, 3D Bioprinting, Hydrogel, and Cardiac Fibrosis surpassed 5 years. Keywords such as Cardiac Microtissues, Energy-metabolism, and Exosomes had burst periods over 4 years. Additionally, Atrial Fibrillation, Personalized Medicine, and Covid-19 demonstrated burst periods greater than 3 years. Notably, in the past three years, Cardiomyocyte Maturation and Cardiac Organoids, with the highest burst strengths of 27.19 and 12.45 respectively, have shown significant citation increases (2022–2024), indicating emerging research frontiers.

#### 3.5.3 Highly cited article analysis

We selected a highly-cited original research article with over 300 citations for analysis. Table 5 presents the top 20 highly-cited citing papers on PSC-CMs from 2007 to 2024. These articles are mainly categorized into five major research themes based on their content: drug discovery and cardiotoxicity assessment, cardiac regenerative therapy, maturation of PSC-CMs, cardiac disease modeling *in vitro* and cardiac tissue engineering. These influential studies, reported by the highly-cited citing articles, have significantly advanced the research of PSC-CMs. Therefore, we will provide a detailed discussion on these studies.

**Table 5:** Top 20 highly cited papers on PSC-CMs during 2007 to 2024.

Rank	Authors	Article title	Journal	Citation	Year
1	Burridge, PW	Chemically defined generation of human cardiomyocytes differentiation	Nat Methods	1,075	2014
2	Chong, JJH	Human embryonic-stem-cell-derived cardiomyocytes regenerate non-humanprimate hearts	Nature	990	2014
3	Ronaldson-Bouchard, K	Advanced maturation of human cardiac tissue grown from pluripotent stem cells	Nature	791	2018
4	Noor, N	3D Printing of Personalized Thick and Perfusable Cardiac Patches and Hearts	Adv Sci	665	2019
5	Wang, G	Modeling the mitochondrial cardiomyopathy of Barth syndrome with induced pluripotent stem cell and heart-on-chip technologies	Nat Med	627	2014
6	Zhang, YS	Bioprinting 3D microfibrous scaffolds for engineering endothelialized myocardium and heart-on-a-chip	Biomaterials	597	2016
7	Khan, M	Embryonic Stem Cell-Derived Exosomes Promote Endogenous Repair Mechanisms and Enhance Cardiac Function Following Myocardial Infarction	Cir Res	571	2015
8	Shiba, Y	Allogeneic transplantation of iPS cell-derived cardiomyocytes regenerates primate hearts	Nature	546	2016
9	Burridge, PW	Human induced pluripotent stem cell-derived cardiomyocytes recapitulate the predilection of breast cancer patients to doxorubicin-induced cardiotoxicity	Nat Med	502	2016
10	Yang, LL	A Human Pluripotent Stem Cell-based Platform to Study SARS-CoV-2 Tropism and Model Virus Infection in Human Cells and Organoids	Cell Stem Cell	466	2020
11	Bassat, E	The extracellular matrix protein agrin promotes heart regeneration in mice	Nature	422	2017
12	Deuse, T	Hypoimmunogenic derivatives of induced pluripotent stem cells evade immune rejection in fully immunocompetent allogeneic recipients	Nat Biotechnol	410	2019
13	Liu, YW	Human embryonic stem cell-derived cardiomyocytes restore function in infarcted hearts of non-human primates	Nat Biotechnol	401	2018
14	Tiburcy, M	Defined Engineered Human Myocardium With Advanced Maturation for Applications in Heart Failure Modeling and Repair	Circulation	393	2017
15	Zhao, Y	A Platform for Generation of Chamber-Specific Cardiac Tissues and Disease Modeling	Cell	363	2019
16	Mathur, A	Human iPSC-based Cardiac Microphysiological System For Drug Screening Applications	Sci Rep	347	2015
17	Wang, YJ	Exosomes/microvesicles from induced pluripotent stem cells deliver cardioprotective miRNAs and prevent cardiomyocyte apoptosis in the ischemic myocardium	Int J Cardiol	329	2015
18	Giacomelli, E	Human-iPSC-Derived Cardiac Stromal Cells Enhance Maturation in 3D Cardiac Microtissues and Reveal Non-cardiomyocyte Contributions to Heart Disease	Cell Stem Cell	315	2020
19	Ruan, JL	Mechanical Stress Conditioning and Electrical Stimulation Promote Contractility and Force Maturation of Induced Pluripotent Stem Cell-Derived Human Cardiac Tissue	Circulation	306	2016
20	Menasché, P	Human embryonic stem cell-derived cardiac progenitors for severe heart failure treatment: first clinical case report	Eur Heart J	304	2015

##### 3.5.3.1 Drug discovery and cardiotoxicity assessment of drugs

Drug cardiotoxicity is a major cause of drug attrition, resulting in numerous preventable patient deaths. The emergence of PSC-CMs offers a promising experimental platform for high-throughput drug testing and the assessment of drug-induced cardiac toxicity. Mathur and colleagues utilized the cardiac microphysiological system (MPS) to precisely forecast cardiotoxicity and determine the effective concentration values of various pharmaceuticals. Their research offers an optimal solution using engineered cardiac tissues, enhancing both drug screening processes and disease modeling capabilities (36). Similarly, Burridge et al. demonstrated the effectiveness of human induced PSC-CMs as an *in vitro* platform for assessing doxorubicin-induced cardiotoxicity and characterizing the genetic foundation and molecular processes underlying doxorubicin-induced cardiotoxicity (37).

##### 3.5.3.2 Cardiac regenerative therapy

Human cardiomyocytes have a significantly low rate of self-renewal, estimated to be approximately 0.3%–1% per year, which is inadequate for restoring normal cardiac structure and function following myocardial infarction. The utilization of PSC-based cardiac regenerative therapy has been extensively studied over the past decade. Out of the 20 articles in Table 5, nine reported on cardiac regenerative therapy. In 2016, Shiba *et al*. first exhibited the efficacy of allogeneic PSC-CM transplantation in regenerating the hearts of nonhuman primates with myocardial infarction. However, there is currently no effective strategy available in clinical practice to repair injured myocardium due to limitations such as a lower engraftment rate, electromechanical uncoupling between grafted cells and resident cells, low purity, and immaturation of differentiated cardiomyocytes. Consequently, researchers have shifted their focuses toward extracellular vesicles or exosomes and have made significant progress in recent years. As early as 2014 (38), Ibrahim et al. suggested that injecting exosomes into damaged mouse myocardium can replicate the regenerative and functional effects achieved through the transplantation of cardiosphere-derived cells (CDCs). Based on these findings, they further proposed that exosomes could be used as cell-free therapeutic candidates for cardiac regeneration, thereby avoiding potential risks associated with CDCs. Subsequently, Wang et al. (39) unveiled that exosomes derived from PSCs can deliver cardioprotective miRNAs and inhibit cardiomyocyte apoptosis in the ischemic myocardium.

##### 3.5.3.3 Maturation of PSC-CMs

The immaturity of PSC-CMs remains a persistent challenge in the field of PSC-CMs. Clearly, it is becoming imperative to address the hurdle that still impedes further clinical applications of PSC-CMs. Among the 20 articles, four specifically focus on the maturation of PSC-CMs.

Early studies focused mostly on the structural maturation of PSC-CMs. In 2013, Kamakura et al. (40) observed that PSC-CMs can relatively mature to acquire more adult-like ultrastructural characteristics over a prolonged culture period. Similarly, Lundy et al. (41) uncovered that extended *in vitro* culture of PSC-CMs can enhance the maturation of their structural and contractile properties to a more adult-like phenotype. While Yang et al. (42) suggested that treatment with triiodothyronine (T3) during cardiac differentiation promotes morphological and structural maturation of PSC-CMs, appearing as elevated sarcoendoplasmic reticulum ATPase levels, increased cardiomyocyte size, anisotropy and sarcomere length. Moreover, when PSC-CMs are cultivated on a matrigel mattress, the addition of thyroid and glucocorticoid hormones to the cardiac differentiation procedure promotes T-tubule formation and matures the excitation-contraction coupling (43).

In recent years, the research focuses have shifted to the functional and metabolic maturation of PSC-CMs. Advanced maturation of PSC-CMs can be achieved when cardiac tissues are subjected to increasing mechanical loading over time (13). Cardiomyocytes normally occupy 75%–80% of the entire volume of myocardium in an adult heart but only account for one-third of the overall cell population, suggesting that non-myocytes play a vital role in maintaining the functional homeostasis of cardiomyocytes (44, 45). As proven by Giacomelli et al. (33), noncardiomyocytes significantly contribute to the functional and metabolic maturation of PSC-CMs in a 3D microtissue that is composed of PSC-CMs, PSC-derived endothelial cells, and cardiac fibroblasts or dermal fibroblasts. This conclusion was further buttressed by other several observations. Important factors contributing to advanced cardiomyocyte maturation in engineered cardiac tissues include the optimal cardiomyocyte-to-nonmyocyte ratio, sequential application of serum-free medium followed by serum-containing medium, low cell seeding density, electromechanical stimulation, composition and stiffness of the extracellular matrix, and cell culture time (typically 7 weeks) (46–48). In a short, based on the timeline distribution of keyword co-occurrence in Figure 7B, it is clear that more studies on the maturity of PSC-CMs, particularly, metabolic maturation, will be conducted in the near future.

##### 3.5.3.4 In vitro **cardiac disease modeling**

*In vitro* cardiac disease modeling using patient-specific PSC-CMs contributes to our comprehensive understanding of the molecular mechanisms underlying the pathogenesis and progression of heart disorder, allowing us to identify potential novel therapeutic targets. Among the top 20 highly cited articles, three are related to this theme. As early as 2013 (49), Kim et al. reported their research on *in vitro* modeling of ARVC/D utilizing patient-specific PSC-CMs. Subsequently, patient-specific PSC-based *in vitro* models for the mitochondrial cardiomyopathy, DCM, and Brugada syndrome were reported sequentially (50–52). Over the past four years, COVID-19 has been declared a global pandemic, causing the deaths of hundreds of thousands of people worldwide. However, the etiology of cardiac injury in COVID-19 remains elusive. Sharma et al. (53) examined the susceptibility of human induced PSC-CMs to infection by SARS-CoV-2 and revealed that SARS-CoV-2 can directly bind to the ACE2 receptor to enter human induced PSC-CMs. Additionally, SARS-CoV-2 can also infect human induced PSC-CMs through extracellular vesicles harboring viral RNA (54). Notably, Yang et al. (55) have established an innovative platform utilizing human cell and organoid models to investigate SARS-CoV-2 tropism. This approach offers a comprehensive understanding of the virus's impact on a variety of cells and tissues, shedding light on the full spectrum of SARS-CoV-2's effects. These work expanded the application of PSC-CMs in identifying potential novel therapeutic targets.

##### 3.5.3.5 Engineered human myocardium

The emergence of microfluidics has significantly advanced the field of cardiac tissue engineering (56). Recently, there has been growing interest in developing the cardiac microphysiological system known as heart-on-a-chip, which has the potential for various applications including personalized cardiac regeneration, drug discovery, cardiotoxicity assessment of drugs and disease modeling. Among the top 20 highly cited papers, two articles have focused on engineered cardiac tissue. Marsano and colleagues had specifically concentrated on generating functional 3D cardiac microtissues using a microfluidic platform and had successfully developed a heart-on-a-chip that can replicate the physiological mechanical environment experienced by cells in the native myocardium (57). Vascularization is essential for the fabrication of engineered cardiac tissues, such as heart-on-a-chip and cardiac organoids, and is the most important factor when recapitulating vascular-based diseases utilizing PSC-based *in vitro* models (58, 59). Zhang et al. (60) constructed an endothelialized myocardium-on-a-chip by adopting an innovative 3D bioprinting microfibrous scaffold to integrate PSC-CMs and vascular endothelial cells together. The model, when combined with a microfluidic perfusion bioreactor, can be used for personalized drug screening. In 2019 (61), Noor et al. further advanced the field by demonstrating an approach to 3D-printed thick, vascularized, and perfusable cardiac patches. However, the vasculature in engineered vascularized tissues still fails to completely mimic the complexity of natural vascular networks due to the poor resolution of 3D bioprinters, which needs to be addressed in the future.

## 4 Discussion

### 4.1 General information

In this study, we analyzed the spatiotemporal distribution of literature, contributions, and collaborations among countries, institutions, and authors in the field of PSC-CMs over the past 18 years. We identified 6,406 papers on PSC-CMs from the WoSCC database, including 5,358 articles and 1,048 reviews, spanning from January 1, 2007, to June 20, 2024. All publications were in English and appeared in 896 academic journals, authored by 29,660 researchers from 81 countries/regions. Moreover, we categorized the original research articles on PSC-CMs according to species-specific PSCs utilizing the SCI-Expanded Web of Science Core Collection filter, revealing that studies on human PSCs accounted for the largest proportion, approximately 40%. Additionally, studies on non-human primate PSCs, pig PSCs, mouse PSCs, rat PSCs, and others were also represented (Supplementary Table S1).

The annual output and citations of literature on PSC-CMs showed an overall uptrend, reaching its peak in 2021, followed by a distinct decline in 2022 and 2023. This decline may be attributed to several challenges posed by the immaturity and heterogeneity of PSC-CMs, as well as concerns regarding tumorigenicity and arrhythmogenicity (19–21). The top 10 prolific nations are located in Europe, North America and Asia. The United States, China, and Germany are the top three major contributors to this area and have the greatest communication and collaboration with one another. The United States contributes approximately 41.90% of the total papers and possesses the highest average citations per article, indicating its dominant position in the field of PSC-CMs. This suggests an imbalance in regional development in the field. Among the top 10 institutions, Stanford University, Harvard University in the United States, and DZHK in Germany are recognized as the leading research centers. It is noteworthy that the top 10 writers with the most papers and citations are mostly from the United States, Germany and Japan, while productive and influential investigators from China remain absent. Circulation Research, Nature and Circulation are three journals with high prestige and significant academic influence in this area based on their highly cited frequencies and impact factors.

### 4.2 An overview of research focuses and frontiers

Organ (heart)-on-a-chip, a prominent hot-topic in recent years, has a 5-year burst period of citations and is attracting significant interest from researchers worldwide because the innovative device bridges the gap between animal models and clinical trials and possesses an extraordinary potential to more accurately assess the cardiotoxicity and efficacy of investigational drugs for clinical application. Recent rapid progress in the field of tissue engineering has made it possible to combine a variety of technologies, including PSC-CMs, multicell-type co-culture, 3D bio-printing, microbio-sensors, microfluidics, and microfabrication engineering. The convergence of multiple technologies has dramatically accelerated the development of cardiac tissue engineering; however, the vascularized 3D cardiac *in-vitro* model is still in its prototype stage and a series of unmet challenges need to be overcome in the near future (62–64).

Maturation of PSC-CMs emerges as the hot-spot keyword with the highest burst strength (27.24) among the 18 keywords with the strongest citation burst from 2018 to 2024. In fact, to date, PSC-CMs still fall short in fully recapitulating the phenotypes of adult cardiomyocytes for disease modeling, cardiotoxicity testing, drug discovery, and regenerative therapy due to their immaturity (13, 65). Various approaches have been attempted to enhance the maturation of PSC-CMs, including long-term culture (41, 66), treatment with T3 (42), mechanical or electrical stimulation (13, 48), treatment with thyroid and glucocorticoid hormones (43), genetic approaches (67), and multicell-type 3D cardiac microtissues (13), However, no single approach has been universally accepted to overcome this barrier (68–70).

Extracellular vesicles (EVs), with a citation burst duration exceeding 6 years, are emerging as a promising tool for the diagnosis and treatment of cardiovascular diseases, including facilitating cardiac regeneration and repair following injury. These nanoscale vesicles harbor a variety of bioactive molecules that modulate recipient cell functions and play critical roles in inflammation, angiogenesis, and tissue repair. Despite their potential, the clinical translation of EVs faces multiple challenges. Firstly, the heterogeneity of EV populations, influenced by their cellular origin and environmental conditions, hinders the development of standardized methods for isolating, characterizing, and ensuring the quality of EVs. Secondly, effective delivery of EVs to cardiac tissue is impeded by their short circulatory half-life and rapid clearance, necessitating enhanced stability and targeted delivery strategies. Thirdly, scaling up the production of clinical-grade EVs while preserving their integrity and function also poses significant manufacturing challenges. Moreover, the mechanisms of EV biogenesis, cargo loading, and uptake by target cells remain poorly understood, impeding the development of targeted EV therapies. Addressing these issues will require concerted efforts from the research community to establish standardized methods, clarify EV functional mechanisms, and innovate delivery techniques, thereby unlocking the full therapeutic potential of EVs for cardiovascular disease management (71–74).

Organoids are 3D, self-organizing structures derived from stem cells that recapitulate key features of their corresponding organs (75). Human cardiac organoids have shown great promise in mimicking the structural and functional properties of the native heart. However, several challenges remain to be addressed before their full potential can be realized, especially in the context of heart disease modeling and treatment. First, current protocols still produce organoids with immature phenotypes compared to adult heart, which limits their utility for modeling adult-onset diseases and developing therapies. Another issue is the lack of standardization in organoid generation. Current protocols often rely on the spontaneous self-assembly of cells, which can lead to variability in organoid size, shape, and cellular composition and hinders reproducibility and comparability across different studies. Third, to fully capture the complexity of the human heart, human cardiac organoids need to incorporate multiple cell types, including cardiomyocytes, endothelial cells, fibroblasts, and immune cells. Achieving the right balance and spatial organization of these cell populations is a challenge for recapitulating the intricate intercellular interactions and signaling pathways that govern cardiac function (69, 70, 76). Fourth, the development of human cardiac organoids that accurately model specific cardiac chambers, such as the atria and ventricles, remains difficult for studying chamber-specific diseases and drug responses (77). Additionally, the absence of a functional vascular network is another limitation, as it leads to suboptimal nutrient and oxygen supply, restricting organoid size and causing cellular heterogeneity (78). Advances in bioengineering, such as the incorporation of supporting cell types, dynamic culture systems, and innovative biomaterials, may help improve the fidelity and maturity of cardiac organoids.

Finally, the global dissemination of COVID-19 imposed a tremendous financial and health burden. SARS-CoV-2 can trigger thromboembolism, myocardial injury, acute coronary syndromes, and arrhythmias in patients with severe cases of COVID-19 (79–81). Currently, there are two proposed mechanisms to explain myocardial injury, but the exact pathophysiological mechanism of cardiac dysfunction in COVID-19 remains elusive (82). In this study, analysis of citation bursts for keywords revealed that COVID-19 and coronavirus have recently experienced a surge in citations (2021–2023), indicating that cardiac injury in COVID-19 will likely become prominent areas of research in the future.

### 4.3 The current status and challenges of clinical trials for stem cell therapy in cardiac diseases

We conducted the search on ClinicalTrials.gov using the terms “heart disease” or “cardiac disease” and “stem cell” to identify 357 relevant clinical trials, with 174 completed trials and 45 involving over 100 participants. However, only 15 clinical trials have published results directly related to stem cell interventions for heart diseases. A summary of these clinical trials is provided in Supplementary Table S2. The primary cardiac conditions targeted in these clinical trials are ischemic cardiomyopathy, myocardial infarction (MI), dilated cardiomyopathy (DCM), left ventricular (LV) dysfunction, and heart failure. Various stem cell types, such as mesenchymal stem cells, bone marrow mononuclear cells, cardiosphere-derived cells, c-kit-positive cardiac cells, bone marrow CD34+ and CD133+ cells, and adipose-derived stem cells, are utilized for the treatment of heart diseases, while the autologous bone marrow-derived mesenchymal stem cells are the most commonly used. Delivery methods for stem cells vary, including transendocardial injection, intracoronary infusion, intramyocardial injection, and intravenous infusion, while transendocardial injection emerges as the preferred approach due to its minimally invasive nature and favorable safety profile. Notably, regarding the clinical application of hiPSC-CMs, to date, no clinical trials involving human induced PSC-CMs for heart disease treatment have been completed, and participant recruitment is still ongoing according to the data from the ClinicalTrials.gov database (Supplementary Table S3), although a case report from Japan documented the successful transplant of allogeneic human induced PSC-CM patches in one patient with ischemic cardiomyopathy [#jRCT2053190081, (83)].

The clinical trials of stem-cell-mediated treatment for heart diseases are at a critical juncture. While these trials have demonstrated safety and potential feasibility, the efficacy of these treatments remains variable and often limited. The heterogeneity and immaturity of stem cells, the challenge of ensuring adequate engraftment and survival post-transplantation, and the timing and method of cell delivery are significant hurdles. Further research is imperative to refine these treatments, with a focus on improving cell maturity, optimizing delivery methods, and understanding the optimal timing for transplantation to enhance the therapeutic potential of stem cells in treating cardiac diseases.

### 4.4 The present study's reliability

To evaluate whether the 6,406 publications retrieved from the Web of Science database are representative for mapping the developmental trajectory of PSC-CM research over the past 18 years, we applied the same retrieval strategy to the PubMed database, yielding 6,363 documents. We re-conducted scientometric analyses, including the co-occurrence and clustering analyses of keywords, and an assessment of author collaborative patterns and author contributions. The results were consistent across both data sets, suggesting our study's reliability (Supplementary Figures S1, S2).

Traditionally, Web of Science and Scopus have been the two most widely used databases for bibliometric analyses, both of which require a subscription. Web of Science is often considered the most user-friendly and accessible tool for bibliometric analysis, leading to its prevalent use in such studies. On the other hand, PubMed, which is freely available and dedicated to biomedical sciences, can optimize the analysis of biomedical subjects. However, the data in NBIB format from the PubMed database lacks references, which makes it challenging to perform comprehensive bibliometric analyses.

### 4.5 Limitation

This study presents a comprehensive analysis of global research on PSC-CMs from a developmental perspective, offering investigators new insights into the current hotspots and emerging trends in PSC-CM research. However, there are still certain limitations. First, we only searched the relevant literature in the WoSCC, PubMed and Clinicaltrials.gov databases, while the pertinent publications in other databases, such as Scopus, might be missed, because existing scientometric software requires the appropriate data format and merging two or more datasets from different database sources for bibliometric analysis is unfeasible. Second, the current bibliometric analysis unavoidably omits some newly published relevant literature; but this omission has little impact on the overall conclusion due to a time lag of citations. In general, recent publications tend to have a relatively low citation frequency. Nonetheless, updating the datasets can enrich the results. Third, the WoSCC, PubMed and Clinicaltrials.gov databases are continuously updated in real time, which means that the publication volume during a specific time period may vary slightly when accessed on different dates. However, given that WoSCC is considered one of the most comprehensive database platforms, PubMed focuses mainly on life sciences and biomedical disciplines and both VOSviewer and CiteSpace are well-established bibliometric software tools, our study should serve as a valuable reference for physicians and researchers in the field.

## 5 Conclusion

The PSC-CM-based *in vitro* cardiac models bridge the gap between animal models and clinical trials, offering vast potential for the translational application of PSC-CMs. This study maps the developmental trajectory of PSC-CM research over the past 18 years to identify hot issues, highlight the developmental trends, and provide a historical perspective to guide scholars in exploring new research directions. From 2007 to 2024, the global annual outputs of literature on PSC-CMs exhibited an overall uptrend, peaking in 2021, with a notable slump in 2022 and 2023. These prominent research hotspots, including fabrication of vascularized and multicell type 3D engineered cardiac models to mimic the intricate natural myocardium, maturation of PSC-CMs, and extracellular vesicles will continue attracting interest from global researchers. Moreover, the exact pathophysiological mechanisms of cardiac involvement in COVID-19, personalized medicine to focus on tailoring treatments to individual patient characteristics and genetic profiles, atrial fibrillation, cardiac fibrosis and energy metabolism of PSC-CMs will be worthy of further exploration in the near future. While the clinical trials of stem-cell-mediated treatment for heart diseases shows promise, significant challenges remain. Further research is imperative to optimize protocols, enhance cell delivery methods, and establish standardized practices to improve clinical outcomes.

## Data Availability

The raw data supporting the conclusions of this article will be made available by the authors, without undue reservation.
