# Supplementary material for: Mapping the landscape of PSC-CM research through bibliometric analysis
Source: Front Cardiovasc Med. 2024 Oct 10;11:1435874. doi: 10.3389/fcvm.2024.1435874 (PMC11499114; doi:10.3389/fcvm.2024.1435874)
Supplement: Supplementary file 1 [file Table1.docx]

| **Table S1** Annual distribution of articles on different species-specific PSCs | | | | | |
| --- | --- | --- | --- | --- | --- |
| **Years** | **mouse-PSCs** | **rat-PSCs** | **pig-PSCs** | **primate-PSCs** | **human-PSCs** |
|  |  |  |  | **(non-human)** |  |
| **2007** | 17 | 9 | 1 | 2 | 32 |
| **2008** | 22 | 0 | 0 | 1 | 35 |
| **2009** | 19 | 4 | 1 | 0 | 41 |
| **2010** | 27 | 7 | 0 | 1 | 52 |
| **2011** | 37 | 4 | 2 | 0 | 66 |
| **2012** | 25 | 6 | 1 | 1 | 63 |
| **2013** | 26 | 2 | 1 | 1 | 104 |
| **2014** | 21 | 3 | 2 | 0 | 99 |
| **2015** | 33 | 6 | 1 | 1 | 115 |
| **2016** | 30 | 3 | 2 | 2 | 124 |
| **2017** | 25 | 6 | 1 | 1 | 163 |
| **2018** | 17 | 9 | 3 | 4 | 169 |
| **2019** | 24 | 3 | 4 | 1 | 198 |
| **2020** | 15 | 8 | 2 | 3 | 205 |
| **2021** | 13 | 9 | 3 | 1 | 234 |
| **2022** | 12 | 6 | 3 | 0 | 223 |
| **2023** | 23 | 6 | 3 | 3 | 152 |
| **2024** | 3 | 2 | 0 | 1 | 67 |
