# Supplementary material for: Mapping the landscape of PSC-CM research through bibliometric analysis
Source: Front Cardiovasc Med. 2024 Oct 10;11:1435874. doi: 10.3389/fcvm.2024.1435874 (PMC11499114; doi:10.3389/fcvm.2024.1435874)
Supplement: Supplementary file 2 [file Table2.docx]

| **Table S2.** Completed clinical trials for stem cell therapy in heart disease. | | | | | | |
| --- | --- | --- | --- | --- | --- | --- |
| **Date** | **NCT Number** | **Study Title** | **Conditions** | **Interventions** | **Population/Phases** | **Conclusion** |
| 2004/4/1-  2009/11/1 | NCT00203203 | Autologous Stem Cells for Cardiac Angiogenesis (FOCUS HF) | Ischemic Cardiomyopathy | Device: Intramyocardial injection of autologous bone marrow mononuclear cells via NOGA Mapping.  Other: Control | 30 participants  age: adult, older adult. sex: all  Phase 1 | Autologous Bone Marrow Mononuclear Cell therapy is safe and improves symptoms, quality of life, and possibly perfusion in patients with chronic HF. |
| 2006/4/1-  2023/8/24 | NCT00314366 | Intramyocardial Injection of Autologous Aldehyde Dehydrogenase-Bright Stem Cells for Therapeutic Angiogenesis (FOCUS Br) | Coronary Artery Disease | Biological:Intramyocardial injection of autologous aldehyde dehydrogenase bright stem cells.  Other: Control (plasma) | 21 participants  age: adult, older adult  sex: all  Phase 1 | We provide preliminary evidence that treatment with the novel cell population, ALDH(br) cells, is safe and may provide perfusion and functional benefits in patients with chronic myocardial ischemia. |
| 2006/5/1-  2013/4/1 | NCT00629018 | Safety and Efficacy Study of Stem Cell Transplantation to Treat Dilated Cardiomyopathy | DCM | Biological: Intracoronary infusion of autologous CD34+ stem cells.  Drug:  Bone marrow stimulation | 110 participants  age: adult, older adult  sex: all  Phase 2 | Intracoronary stem cell transplantation could lead to improved ventricular remodeling, better exercise tolerance and potentially improved survival in patients with DCM. |
| 2008/7/1-  2012/11/1 | NCT00684021 | Use of Adult Autologous Stem Cells in Treating People Who Have Had a Heart Attack (The TIME Study) | LV Dysfunction | Biological:  Intracoronary infusion of adult bone marrow mononuclear cells  Biological: Placebo | 120 participants  age: adult, older adult  sex: all  Phase 2 | The clinical trial results indicate that administering intracoronary bone marrow mononuclear cells (BMCs) at 3 or 7 days post-ST-segment-elevation myocardial infarction (STEMI) shows no significant improvement in global or regional left ventricular (LV) function compared to a placebo. Using cardiac magnetic resonance imaging, it was observed that microvascular obstruction correlates with poorer LV function recovery, adverse LV remodeling, and higher device implantation rates. The study highlights that baseline BMC characteristics are more critical for myocardial repair than the timing of BMC transplantation. Additionally, specific BMC features, such as endothelial precursor activity and CD34+ cell percentages, were linked to better clinical outcomes, supporting targeted selection of potent cell subsets for therapy. |
| 2008/7/1-  2012/2/1 | NCT00684060 | Use of Adult Autologous Stem Cells in Treating People 2 to 3 Weeks After Having a Heart Attack (The Late TIME Study) | LV Dysfunction | Biological:  Intracoronary infusion of adult bone marrow CD34+ and CD133+ stem cells.  Biological: Placebo | 87 participants  age: adult, older adult  sex: all  Phase 2 | The study reveals that the bone marrow (BM) cell composition and its subsets, particularly CD34+ and CD11b cells, influence recovery after acute myocardial infarction (AMI) and subsequent cell therapy. Greater BM CD34+ and CD133+ cell abundance correlates with improved left ventricular function, while higher CD11b(dim) cells correlate with poorer outcomes. The study also suggests that blocking IL-1 or IL-6 receptors can enhance BM regenerative capacity. Despite these findings, intracoronary infusion of autologous BM cells did not significantly improve heart function in clinical trials compared to placebo. |
| 2008/8/1-  2013/9/1 | NCT00768066 | The Transendocardial Autologous Cells (hMSC or hBMC) in Ischemic Heart Failure Trial (TAC-HFT) | Stem Cell Transplantation  LV Dysfunction | Biological:  Transendocardial injection of autologous hMSCs and hBMCs.  Biological: Placebo | 65 participants  age: adult, older adult  sex: all  Phase 1&2 | Transendocardial stem cell injection with MSCs or BMCs appears safe for chronic ischemic cardiomyopathy patients with LV dysfunction, though larger studies are needed for definitive evidence on safety and efficacy. Mesenchymal stem cell therapy benefits both DCM and ICM patients by enhancing quality of life and functional capacity, with DCM patients showing improved cardiac function and ICM patients experiencing reverse remodeling. |
| 2009/3/1-  2012/5/1 | NCT00824005 | Effectiveness of Stem Cell Treatment for Adults With Ischemic Cardiomyopathy (The FOCUS Study) | Chronic Ischemic Heart Disease,  LV Dysfunction  Angina, Ischemic Cardiomyopathy | Biological: Intramyocardial injection of adult autologous bone marrow mononuclear cells.  Biological: Placebo | 92 participants  age: adult, older adult  sex: all  Phase 2 | The clinical trial aimed to evaluate the effectiveness of autologous bone marrow mononuclear cells (BMCs) in improving heart function among patients with CAD and LV dysfunction. Despite thorough monitoring over five years, results showed that BMC therapy did not significantly enhance left ventricular end-systolic volume (LVESV), maximal oxygen consumption, or SPECT reversibility compared to placebo. The trial identified variability in BM cell subsets and correlations between specific cell populations and heart function improvements, suggesting these factors may contribute to the modest clinical benefits observed in previous BMC therapy studies. This emphasizes the need for selecting potent cell subsets and addressing bone marrow impairments. |
| 2011/1/1-  2013/1/1 | NCT01273857 | Transcoronary Infusion of Cardiac Progenitor Cells in Patients With Single Ventricle Physiology | Hypoplastic Left Heart Syndrome,  Single Ventricle  Heart Failure | Procedure: Transcoronary infusion of autologous cardiosphere-derived cells.  Procedure: staged shunt procedure | 14 participants  age: child  sex: all  Phase 1 | Clinical trials investigating the use of cardiosphere-derived cells (CDCs) in various cardiac conditions have shown promising results. These studies indicate that CDC therapy is generally safe and feasible, particularly in children with hypoplastic left heart syndrome and patients with single ventricles. The treatment has demonstrated improvements in ventricular function, reduced complications, and enhanced somatic growth. Additionally, controlled delivery of growth factors has shown potential in improving CDC engraftment and differentiation, leading to functional improvements after myocardial infarction. While these findings are encouraging, larger phase 2 trials are necessary to further evaluate long-term benefits and clinical outcomes, especially in patients with heart failure. |
| 2011/5/19-2017/8/28 | NCT01392625 | PercutaneOus StEm Cell Injection Delivery Effects On Neomyogenesis in Dilated CardioMyopathy (The POSEIDON-DCM Study) | Non-ischemic DCM | Biological: Intramyocardial injections of autologous hMSCs  Biological: Intramyocardial injections of allogeneic hMSCs | 37 participants  age: adult, older adult  sex: all  Phase 1&2 | The clinical trial results indicate that transcatheter intramyocardial injections of autologous bone marrow progenitor cells improve regional contractility in chronic myocardial scars, potentially leading to reverse remodeling. The study found both safety and efficacy in using allo-hMSCs versus auto-hMSCs in NIDCM patients, suggesting the need for further trials. Additionally, MSC therapy shows benefits in DCM and ICM patients, enhancing quality of life and functional capacity, with variable impacts on cardiac phenotypes across patient groups. |
| 2012/4/1-  2015/11/1 | NCT01508910 | Efficacy and Safety of Targeted Intramyocardial Delivery of Auto CD34+ Stem Cells for Improving Exercise Capacity in Subjects With Refractory Angina | Chronic MI Refractory Angina Pectoris,  Advanced Coronary Heart Disease | Biological: Intramyocardial injection of auto-CD34+ cells. Biological: Placebo: Diluent used to suspend Auto-CD34+ cells  OTHER: Standard of care | 291 participants  age: adult, older adult  sex: all  Phase 3 | Due to early termination, RENEW was an incomplete experiment; however, the results were consistent with observations from earlier phase studies. These findings underscore the need for a definitive trial. |
| 2014/2/13-2017/9/18 | NCT02013674 | The TRansendocardial Stem Cell Injection Delivery Effects on Neomyogenesis STudy (The TRIDENT Study) | Chronic Ischemic  LV Dysfunction  MI | Biological: Transendocardial injection of allogeneic hMSCs | 30 participants  age: adult, older adult  sex: all  Phase 2 | Although both cell doses reduced scar size, only the 100 million dose increased ejection fraction. This study highlights the crucial role of cell dose in the responses to cell therapy. Determining optimal dose and delivery is essential to advance the field, decipher mechanism(s) of action and enhance planning of pivotal Phase III trials. |
| 2014/6/1-  2017/5/11 | NCT02467387 | A Study to Assess the Effect of Intravenous Dose of aMBMC to Subjects With Non-ischemic Heart Failure | Non-Ischemic  Heart Failure | Drug: Intravenous infusion of allogeneic mesenchymal bone marrow cells (aMBMC). Drug:  Lactated ringer's solution | 23 participants  age: adult, older adult  sex: all  Phase 2 | In this pilot study of patients with nonischemic cardiomyopathy, MSC therapy was safe, caused immunomodulatory effects, and was associated with improvements in health status and functional capacity. |
| 2015/10/1-2020/7/22 | NCT02501811 | Combination of Mesenchymal and C-kit+ Cardiac Stem Cells as Regenerative Therapy for Heart Failure | Ischemic Cardiomyopathy | Biological:  Transendocardial injection of MSCs.  Biological: c-kit+ cells  Biological: Placebo (Plasmalyte A) | 125 participants  age: adult, older adult  sex: all  Phase 2 | The CONCERT-HF trial aims to evaluate the therapeutic potential of autologous bone marrow-derived mesenchymal stem cells (MSCs) and C-kit+ cardiac progenitor cells (CPCs), individually and combined, in treating HF due to ischemic cardiomyopathy, using ECV-guided LGE for scar quantification. |
| 2016/8/1-  2020/4/20 | NCT02509156 | Stem Cell Injection in Cancer Survivors | Cardiomyopathy Due to Anthracyclines | Biological: Transendocardial injection of allo-MSCs  Biological: Placebo | 46 participants  age: adult, older adult  sex: all  Phase 1 | Efficient cell therapy delivery for NICM can be achieved via electromechanical navigation, with immunoselected MPCs showing benefits for nonischemic heart conditions. SENECA, the first clinical trial using direct cardiac cell injection for AIC treatment, aims to establish the feasibility and safety of allogeneic mesenchymal stem cells (allo-MSCs), potentially leading to larger phase II/III studies focused on therapeutic efficacy. |
| 2017/4/14-2020/12/9 | NCT03129568 | Transcoronary Infusion of Cardiac Progenitor Cells in Pediatric Dilated Cardiomyopathy | DCM | Biological:  Transcoronary infusion of cardiosphere-derived cells (CDCs) | 5 participants  age: child  sex: all  Phase 1 | The clinical trial results suggest that intracoronary infusion of cardiosphere-derived cells (CDCs) post-staged palliation in patients with single ventricle physiology, such as those with hypoplastic left heart syndrome (HLHS), is safe and beneficial. The treatment contributed to reverse cardiac remodeling, improved right ventricular ejection fraction (RVEF), and potentially enhanced somatic growth and quality of life while reducing heart failure and parental stress. These positive effects were observed to persist over a 36-month follow-up period. The findings support further large phase 2 trials to validate the long-term clinical benefits. |
