# Supplementary material for: Mapping the landscape of PSC-CM research through bibliometric analysis
Source: Front Cardiovasc Med. 2024 Oct 10;11:1435874. doi: 10.3389/fcvm.2024.1435874 (PMC11499114; doi:10.3389/fcvm.2024.1435874)
Supplement: Supplementary file 3 [file Table3.docx]

| **Table S3** The clinical trials of hiPSC-CM-meidated treatment for heart disease (Participants recruiting is ongoing) | | | | | | | |
| --- | --- | --- | --- | --- | --- | --- | --- |
| **Start Date** | **NCT Number** | **Study Title** | **Study Status** | **Conditions** | **Interventions** | **Phases** | **Brief Summary** |
| 2023/2/3 | NCT05647213 | Autologous iPSCs of Cardiac Lineage for Congenital Heart Disease | Recruiting | Univentricular Heart  Congenital Heart Disease  Heart Failure NYHA Class III  Heart Failure NYHA Class IV | Biological: iPSC-CL | Phase1 | The goal of this clinical trial is to test the safety of lab-grown heart cells made from stem cells in subjects with congenital heart disease. |
| 2022/10/9 | NCT05566600 | Allogeneic iPSC-CM Therapy in Patients With Worsening Ischemic Heart Failure | Recruiting | Ischemic Heart Failure  Chronic Heart Failure | Biological: allogeneic hiPSC-CMs | Early phase1 | This study is designed to evaluate the safety and efficacy of allogeneic iPSC-CMs in treating patients with worsening ischemic heart failure undergoing coronary artery bypass graft surgery. After screening, iPSC-CMs will be administrated intramyocardially in consented and eligible patients undergoing open-chest CABG surgery and the estimated population size for the study will be 32 patients. |
| 2022/4/19 | NCT04945018 | A Study of iPSC-CM Spheroids (HS-001) in Patients With Heart Failure (LAPiS Study) | Recruiting | Heart Failure  Ischemic Heart Disease | Biological:  HS-001 CS  DEVICE:  HS-001-D needle, HS-001-D Adaptor | Phase1&2 | The purpose of this clinical study is to evaluate the safety and efficacy of HS-001 CS transplanted into severe heart failure patients with underlying ischemic heart disease for 26 weeks after transplantation. |
| 2022/6/30 | NCT05223894 | Treating Heart Failure With hiPSC-CMs | Recruiting | Heart Failure | Biological: hiPSC-CM  OTHER: Control | Phase1&2 | The purpose of this study is to assess the safety and efficacy of intramyocardial delivery of hiPSC-CMs at the time of coronary artery bypass grafting in patients with chronic heart failure. |
| 2023/9/5 | NCT06340048 | Epicardial Injection of hiPSC-CMs to Treat Severe Chronic Ischemic Heart Failure | Recruiting | Heart Failure | DRUG:  HiCM-188 therapy | Phase1&2 | The purpose of this clinical study is to evaluate the feasibility, safety and efficacy of intramyocardial injection of hiPSC-CMs (HiCM-188) during coronary artery bypass grafting (CABG) surgery in patients with severe chronic ischemic heart failure. |
| 2021/10/8 | NCT03763136 | Treating Heart Failure With hPSC-CMs | Recruiting | Heart Failure | Biological: hPSC-CM therapy | Phase1&2 | The purpose of this study is to assess the safety, feasibility and efficacy of intramyocardial delivery of cardiomyocytes at the time of coronary artery bypass grafting in patients with chronic heart failure. |
| 2020/2/3 | NCT04396899 | Safety and Efficacy of iPSC-derived Engineered Human Myocardium as Biological Ventricular Assist Tissue in Terminal Heart Failure | Recruiting | Heart Failure | Biological:  EHM implantation | Phase1&2 | The BioVAT-HF trial will test the hypothesis that cardiomyocyte implantation via engineered heart muscle (EHM), the proposed investigational medicinal product (IMP; designated "Biological Ventricular Assist Tissue" or BioVAT), results in sustainable remuscularization and Biological enhancement of myocardial performance in the failing heart. EHM are constructed from defined mixtures of iPSC-CMs and stromal cells in a bovine collagen type I hydrogel. Comprehensive preclinical testing confirmed the rationale for the clinical translation of the myocardial remuscularization strategy by EHM implantation. The patient target population for EHM therapy is patients suffering from advanced heart failure with reduced ejection fraction (HFrEF; EF: ≤35%) and no realistic option for heart transplantation. |
| 2021/9/21 | NCT04982081 | Treating Congestive HF With hiPSC-CMs Through Endocardial Injection | Recruiting | Cardiovascular Diseases  Congestive Heart Failure  DCM | Biological: hiPSC-CM therapy | Phase1 | The main purposes of this explanatory study is to investigate the safety and efficacy of the catheter-based endocardial delivery of human iPSC-CMs in patients with congestive heart failure. |
