# Supplementary material for: Mapping the landscape of PSC-CM research through bibliometric analysis
Source: Front Cardiovasc Med. 2024 Oct 10;11:1435874. doi: 10.3389/fcvm.2024.1435874 (PMC11499114; doi:10.3389/fcvm.2024.1435874)
Supplement: Supplementary file 5 [file Image2.pdf]

Supplementary Figure S2

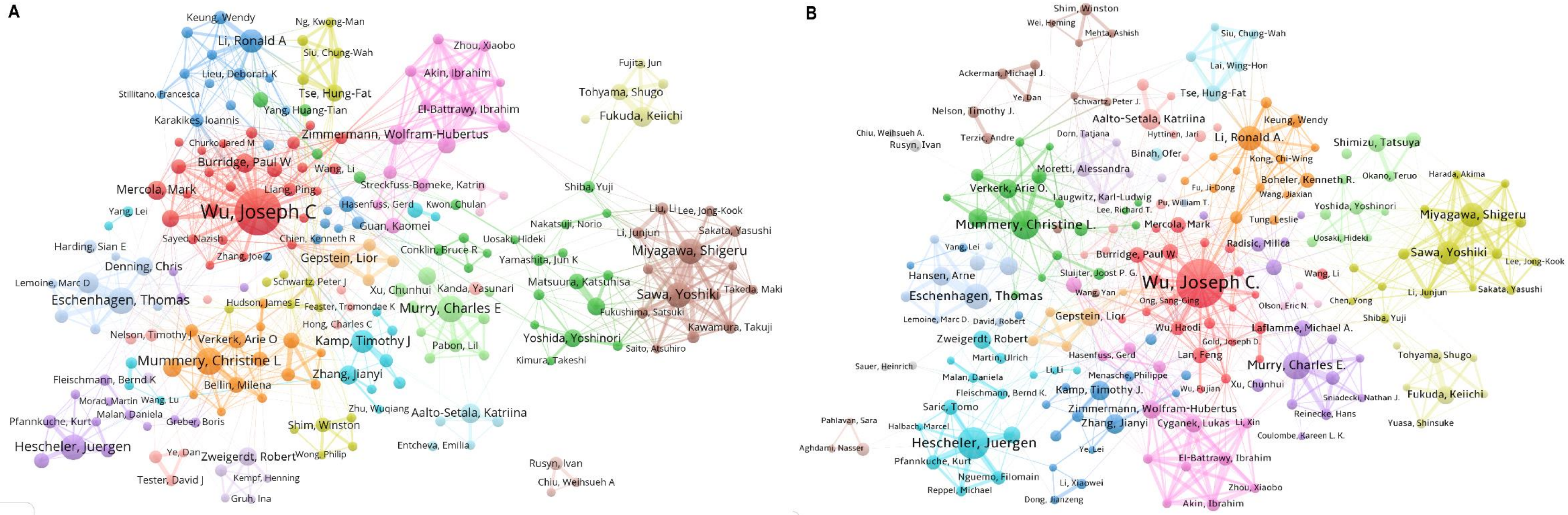

Supplementary Figure S2 Collaboration and contribution analysis of authors reveals a consistent distribution across both datasets derived from the PubMed and Web of Science (WoS) databases. (A) The collaboration and contribution pattern of authors based on the datasets from the PubMed database. (B) The collaboration and contribution pattern of authors based on the datasets from the WoS database. Author collaboration network based on the co-authorship of papers related to PSC-CMs (articles per author ⩾ 15). The sizes of nodes correspond to the number of papers. The links indicate the existence of a cooperative relationship among the authors, with the thicker line corresponding to a closer partnership. Authors within the same cluster exhibit a higher degree of collaboration.
