# Supplementary material for: Mapping the landscape of PSC-CM research through bibliometric analysis
Source: Front Cardiovasc Med. 2024 Oct 10;11:1435874. doi: 10.3389/fcvm.2024.1435874 (PMC11499114; doi:10.3389/fcvm.2024.1435874)
Supplement: Supplementary file 4 [file Image1.pdf]

**Supplementary Figure S1**

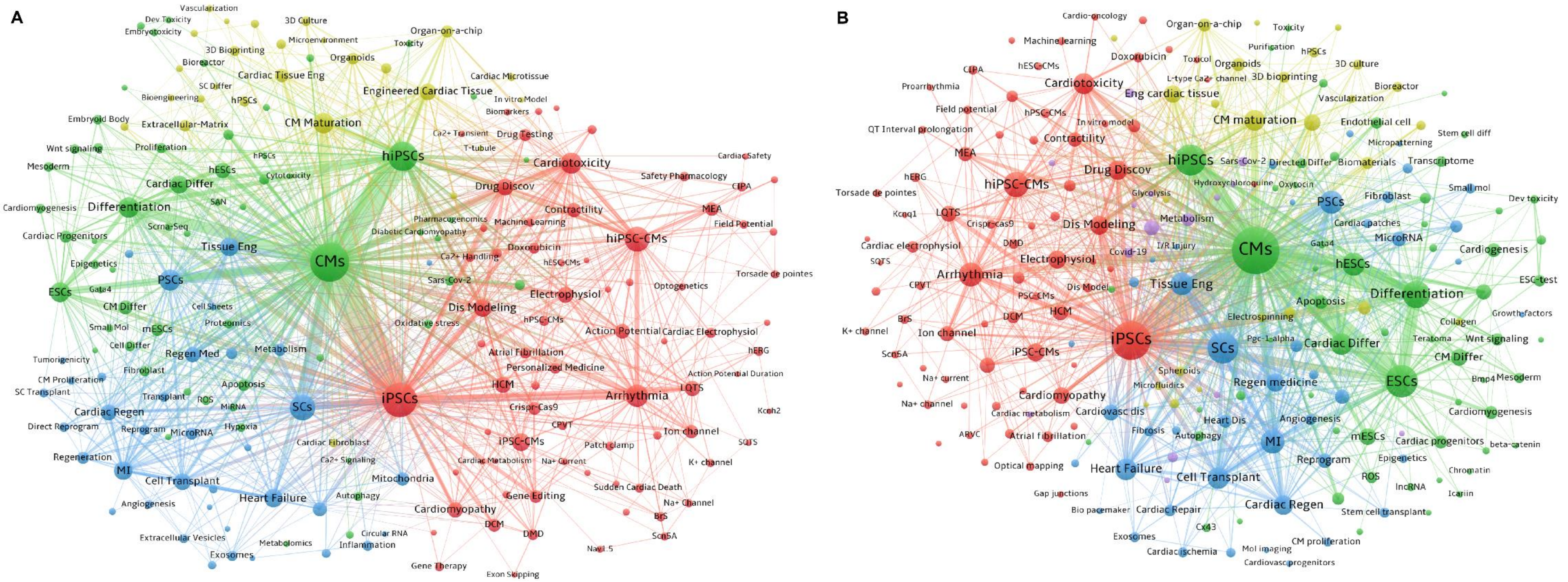


**Supplementary Figure S1** The clustering analysis of keywords demonstrates a consistent clustering pattern across both datasets derived from the PubMed and Web of Science (WoS) databases**.** (A) Keyword Clustering pattern based on the datasets from the PubMed database. (B) Keyword Clustering pattern based on the datasets from the WoS database. Each node represents a keyword. The size of the node indicates the occurrence frequency of a keyword, and the larger the node is, and the higher its corresponding occurrence frequency. Keywords with close correlation are grouped into one cluster with the same color. The thickness of the connecting line between nodes reflects the strength of co-occurrence, with a thicker line indicating a higher co-occurrence frequency of the two connected keywords.
